# A Revised Classification Framework for Studies in Systematic Reviews: Prioritizing Outcome Data Availability Over Study Status

**DOI:** 10.1111/jep.70531

**Published:** 2026-08-06

**Authors:** Rafael Leite Pacheco, Rachel Riera

**Affiliations:** ^1^ Health Technology Assessment Center Hospital Sírio‐Libanês São Paulo Brazil; ^2^ Discipline of Evidence‐Based Medicine, Escola Paulista de Medicina Universidade Federal de São Paulo (Unifesp) São Paulo Brazil; ^3^ Departamento de Medicina Centro Universitário São Camilo São Paulo Brazil

**Keywords:** research synthesis, study category, study classification, systematic reviews

## Abstract

**Background:**

The classification of primary studies is a critical methodological step in the conduct of systematic reviews. The current Cochrane approach categorizes studies as ‘included’, ‘excluded’, ‘ongoing’ or ‘awaiting classification’, primarily based on study eligibility and completion status. However, the concept of an ‘ongoing’ clinical trial is itself poorly defined across registries and methodological guidelines, introducing a layer of subjectivity that undermines reproducibility.

**Methods:**

We propose a revised classification framework that replaces study status with outcome data availability as the primary classification criterion. Results: The proposed framework introduces three changes: (1) splitting the ‘included studies’ category into ‘included studies with analysable outcome data‘ and ‘included studies without analysable outcome data’; (2) eliminating the ‘ongoing studies’ category, whose function is absorbed by the two proposed categories; and (3) restricting ‘awaiting classification’ exclusively to studies where eligibility could not be determined.

**Conclusion:**

This framework makes more explicit how many eligible studies could not contribute to outcome analysis due to reporting‐related problems, thereby increasing transparency and reducing ambiguity in the categorization process. Future empirical studies measuring inter‐rater reliability and the operational precision of the proposed categories are needed to validate this proposal.

1

The proper classification of primary studies is a critical methodological step when planning and conducting a systematic review. The Cochrane Collaboration recommends a four‐category approach comprising: (1) included studies, (2) excluded studies, (3) ongoing studies and (4) awaiting classification studies [1]. This framework is organized around two main dimensions: study eligibility and study completion status.

According to this approach, included studies are those that are completed and fulfil the review's eligibility criteria, regardless of publication language, format or outcome data availability. In contrast, excluded studies are those that clearly do not fulfil the eligibility criteria. Ongoing studies are those that satisfy eligibility criteria but have not been completed by the date of the review, with the potential to be incorporated in future updates. The fourth category, awaiting classification, is recommended by item C37 of the Methodological Expectations of Cochrane Intervention Reviews (MECIR) as a repository for potentially relevant studies that have been conducted and are known to the review team, but have not yet been incorporated into the review [2]. However, because MECIR provides no additional guidance on what aspects should or should not determine this classification, review authors often apply empirical criteria and the justifications for categorizing a study as ‘awaiting’ are frequently ambiguous or underreported [3].

In a previous study, we analysed 260 Cochrane reviews published in the first half of 2019, documenting the reasons stated by authors for classifying studies as ‘awaiting classification’. Among 426 such studies, we identified justifications that were misleading or inadequate when presented without further explanation, including ‘study completed but without published results’ (15%), ‘full‐text unavailable’ (14.5%), ‘ongoing study/clinical trial registry of an RCT’ (8.9%) and ‘population of interest was a subgroup of the analysis’ (8.45%) [3].

A further source of ambiguity in the current framework concerns the definition of ‘ongoing’ itself. A cross‐sectional analysis of clinical trial registries, methodological guidelines and other relevant sources found heterogeneous definitions for both the start and end of a clinical trial [4]. Notably, some sources, including the International Committee of Medical Journal Editors, consider a study to still be ‘ongoing’ during the data analysis phase, meaning that completion of data collection does not unambiguously mark the end of a study. This definitional heterogeneity means that classification based on ‘ongoing’ status introduces an additional, poorly controlled layer of subjectivity, further undermining the consistency and reproducibility of the categorization process.

We propose a revised framework that addresses these problems by replacing study status with *outcome data availability* as the primary classification criterion, alongside study eligibility (Table 1). The central rationale is that an eligible ongoing study with published analysable outcome data is functionally closer to a completed study with analysable data than to an ongoing study that contributes nothing to the analysis. Conversely, a completed study with unpublished or unobtainable data is operationally equivalent to a study whose evidence is not yet available, irrespective of its formal completion status.

**Table 1:** Comparison of the current Cochrane approach and the proposed revised framework for categorizing studies in systematic reviews.

Current Cochrane approach	Proposed revised framework
Included studies	*Included studies with analysable outcome data*
Studies that fulfil all eligibility criteria and are completed.	Studies that fulfil all eligibility criteria AND have analysable/usable outcome data available.
Encompasses studies regardless of outcome data availability.	Study completion status is not a determining criterion.
Ongoing studies	*Included studies without analysable outcome data*
Studies that fulfil all eligibility criteria but are not completed.	Studies that fulfil all eligibility criteria but do NOT have analysable/usable outcome data available.
*Note: the definition of ‘ongoing' varies across registries and guidelines*.	Absorbs both currently ‘ongoing’ studies without data and completed studies with unavailable/unusable data.
Excluded studies	*Excluded studies*
Studies that do not fulfil eligibility criteria.	Studies that do not fulfil eligibility criteria, regardless of status or outcome data availability.
Awaiting classification studies	*Awaiting classification studies*
Potentially relevant studies not yet incorporated in the review (broad, poorly defined criterion).	Studies where a judgement regarding eligibility was NOT possible at the time of the review (restricted, operationally defined criterion).

The main structural changes in the proposed framework are: (1) splitting ‘included studies’ into *included studies with analysable outcome data* and *included studies without analysable outcome data*; (2) eliminating the ‘ongoing studies’ category, whose informational function is fully absorbed by these two proposed categories; and (3) restricting ‘awaiting classification’ exclusively to studies where a judgement regarding eligibility was not possible at the time of the review. Illustrative examples for each category are provided in Table 2.

**Table 2:** Examples for each proposed category.

Category	Examples
Included studies with analysable outcome data	Completed studies with analysable/usable outcome data (full‐text, abstract or any other format). Ongoing studies with preliminary results providing analysable outcome data. Completed/ongoing studies where the population of interest is a subgroup, and data were presented separately enabling outcome analysis. Studies with missing outcome data where imputation methods were applied and outcome analysis was possible.
Included studies without analysable outcome data	Completed studies with no analysable/usable outcome data (in any format). Ongoing studies with no published or obtainable outcome data. Studies with outcome data presented only in a format not pre‐specified in the review protocol. Studies with missing outcome data of an extent that prevented outcome analysis. Studies where the population of interest is a subgroup and data were not presented separately, preventing outcome analysis. Studies whose full report is available in a language or format that prevented outcome data extraction.
Excluded studies	Studies that did not fulfil the eligibility criteria, regardless of completion status or outcome data availability.
Awaiting classification studies	Studies without sufficient information to decide about eligibility, including poorly reported manuscripts, studies published only as abstracts or conference proceedings, clinical trial registry records with insufficient detail and untranslated studies where a judgement about eligibility criteria was not possible.

The elimination of the ‘ongoing’ category warrants explicit justification. Under the current approach, this category signals to readers that additional evidence is forthcoming, serving a prospective informational function. The proposed framework preserves this function: an ongoing study without available data is classified as ‘included without analysable outcome data’, and the accompanying study table should specify the study's ongoing status, attempts to retrieve outcome data, an estimate of when data may become available and a discussion of how inclusion of those data might change the review's conclusions.

The proposed category ‘included studies without analysable outcome data’ makes explicit the number of eligible studies that could not contribute to the outcome analysis due to reporting‐related problems, a dimension that is currently obscured by the conflation of eligibility uncertainty and data unavailability within the ‘awaiting’ category.

We recognize that the boundary between ‘analysable’ and ‘non‐analysable’ data can itself be a source of subjectivity. Review authors should therefore operationalize this criterion explicitly in their protocol, specifying in advance the minimum data requirements for a study to qualify as ‘with analysable outcome data’ (e.g., availability of means and measures of dispersion, or event counts and denominators). Cases where data are partially available but imputation methods were applied should be classified as ‘with analysable outcome data’, provided the imputation is documented.

It is important to emphasize that the proposed recategorization does not alter the methodology for evidence analysis or effect estimation. The MECIR already specifies that a study should not be excluded solely because outcome data are not reported in a ‘usable way’ (item C40) [2], and item C8 discourages authors from adopting outcome availability as a sole eligibility criterion [2]. Nevertheless, empirical evidence suggests that many systematic reviews continue to handle eligible studies with unavailable outcome data inconsistently, either excluding them or failing to identify them explicitly [5]. The proposed framework directly addresses this gap by creating a distinct, mandatory category for such studies, making visible a dimension of evidence completeness that is currently obscured. The proposed recategorization is fully consistent with MECIR requirements and does not alter the methodology for evidence analysis or effect estimation.

We recognize that some review authors choose to make outcome availability an eligibility criterion in specific circumstances (e.g., when an intervention serves different clinical purposes). In those cases, the category ‘included without analysable outcome data’ would not be applicable, which is acceptable provided the rationale is pre‐specified in the protocol.

Regarding reporting standards, the PRISMA 2020 statement distinguishes studies included in qualitative synthesis from those included in quantitative synthesis (meta‐analysis) [6]. The proposed category ‘included with analysable outcome data’ maps onto any study that contributed to the overall outcome analysis, not exclusively to meta‐analysis, making it relevant for qualitative synthesis methods as well. Future revisions of PRISMA guidance could consider accommodating the proposed categories to facilitate implementation.

It is important to note that study status and outcome data availability are independent dimensions. A study classified as recruiting, completed but unpublished, terminated early or suspended may or may not provide analysable outcome data, and any of these statuses are compatible with both *included studies with analysable outcome data* and *included studies without analysable outcome data*; categories. Because the two dimensions are independent they should not be collapsed into a single classification and, therefore, the proposed framework therefore does not discard study status. Outcome data availability becomes the primary criterion, alongside eligibility, because it determines whether a study contributes to the analysis, while study status is preserved as a mandatory descriptor that also should be reported. This arrangement makes explicit how many eligible studies in fact contribute to outcome‐level analysis, regardless of their formal status and retains the prospective and methodological information that status conveys, including the distinction between a study whose data may still appear and one whose results exist but cannot be obtained.

A key limitation of the proposed framework concerns the level at which outcome data availability operates. Availability is a property of a specific outcome within a study rather than of the study as a whole, since an eligible study may provide analysable data for one outcome and not for another, and empirical evidence indicates that this pattern is common rather than exceptional [7]. The binary study‐level labels ‘with’ and ‘without analysable outcome data’ cannot, on their own, capture this heterogeneity. We therefore intend the classification to be applied with reference to the review's pre‐specified outcome set, so that a study is classified as ‘with analysable outcome data’ when it provides usable data for at least one outcome of interest, while the outcomes that could and could not be analysed are recorded for each study, ideally through an outcome availability matrix. Under this reading, the proposed categories indicate whether a study contributes to any part of the synthesis, and the outcome‐level detail required for interpretation is preserved in the accompanying study table rather than collapsed into the category label.

In conclusion, the definition and application of study categories in systematic reviews require greater clarity and operational rigour. We propose a revised framework grounded in outcome data availability rather than study status, supported by empirical evidence documenting the ambiguity of both the current ‘awaiting’ category [3] and the concept of ‘ongoing’ status itself [4]. Future studies measuring inter‐rater reliability and the operational precision of the proposed categories are needed to validate and refine this proposal.

## 2 Practical Application: Reclassification of a Published Cochrane Review

To illustrate how the proposed framework would operate in practice, we applied it retrospectively to a published Cochrane review (Table 3) [8]. This review was selected because it exemplifies a common and methodologically instructive pattern: a relatively large number of included studies coexisting with substantial heterogeneity in data availability and study status, and a diverse set of reasons for classifying studies as ‘awaiting classification’ or ‘ongoing’.

**Table 3:** Study‐by‐study reclassification of the ‘awaiting classification’ and ‘ongoing’ studies from Smart et al. [6] under the proposed framework.

Study	Current classification	Reason reported by authors	Eligibility confirmable?	Outcome data available?	Proposed classification
Dimitrijevic 2019	Awaiting classification	Published as conference abstract only	Conditional^a^	No	If eligible confirmed by information on the abstract: Included without analysable outcome data; if not: Awaiting classification
Dimitrijevic 2020	Awaiting classification	Published as conference abstract only	Conditional^a^	No	If eligible confirmed by information on the abstract: Included without analysable outcome data; if not: Awaiting classification
Mallikarjunaiah 2015	Awaiting classification	Published as conference abstract only	Conditional^a^	No	If eligible confirmed by information on the abstract: Included without analysable outcome data; if not: Awaiting classification
Patru 2017	Awaiting classification	Published as conference abstract only	Conditional^a^	No	If eligible confirmed by information on the abstract: Included without analysable outcome data; if not: Awaiting classification
ISRCTN39729827	Awaiting classification	Authors not contactable; study status unknown	Conditional^a^	No	If eligible confirmed by registry: Included without analysable outcome data; if not: Awaiting classification
ISRCTN97144266	Awaiting classification	Authors not contactable; study status unknown	Conditional^a^	No	If eligible confirmed by registry: Included without analysable outcome data; if not: Awaiting classification
NCT01944150	Awaiting classification	Authors not contactable; study status unknown	Conditional^a^	No	If eligible confirmed by registry: Included without analysable outcome data; if not: Awaiting classification
UKCRN ID 12602	Awaiting classification	Trial delayed; outcome data unavailable	Yes	No	Included without analysable outcome data
NCT03377504	Ongoing	Completed; data being analysed	Yes	No	Included without analysable outcome data
NCT03887962	Ongoing	Completed; data being analysed	Yes	No	Included without analysable outcome data
NCT02395211	Ongoing	Actively ongoing; no results published	Yes	No	Included without analysable outcome data
NCT02753335	Ongoing	Actively ongoing; no results published	Yes	No	Included without analysable outcome data
JPRN‐UMIN000029801	Ongoing	Trial delayed; no results	Yes	No	Included without analysable outcome data
ChiCTR1900020835	Ongoing	Progress unknown; no results	Yes	No	Included without analysable outcome data
CTRI/2019/01/017272	Ongoing	Progress unknown; no results	Yes	No	Included without analysable outcome data

^a^ Eligibility may be confirmable without direct author contact, depending on the details available in the trial registration record or in the conference abstract.

The review included 34 RCTs (1339 participants). Due to the diversity of physiotherapy interventions tested and outcome measures used, the authors were unable to pool results across trials; therefore, each comparison was based on single trials or small groups of trials reported narratively. Eight studies were classified as ‘awaiting classification’ and seven as ‘ongoing’.

Under the proposed framework, the 34 included studies would be split into two subcategories. Studies that contributed data to the outcome analyses, regardless of whether they were formally completed, would be classified as *included studies with analysable outcome data*. Studies that met eligibility criteria but from which no outcome data could be extracted.

The reclassification of the eight ‘awaiting classification’ studies is detailed in Table 3 and is particularly instructive. Seven of the eight studies have a conditional proposed classification that depends on whether eligibility can be established from available information. Four were published only as conference abstracts (Dimitrijevic 2019; Dimitrijevic 2020; Mallikarjunaiah 2015; Patru 2017): if the abstract provides sufficient information to confirm eligibility, these would be reclassified as *included without analysable outcome data*; if eligibility cannot be assessed from the abstract, they appropriately remain as *awaiting classification*. Three further studies remained ‘awaiting’ because the review authors were unable to contact the investigators (ISRCTN39729827; ISRCTN97144266; NCT01944150); the same conditional logic applies: if eligibility can be confirmed from registry data, these move to *included without analysable outcome data*; otherwise, they remain as *awaiting classification*. The only unambiguous case among the eight is a trial that was delayed (UKCRN ID 12602): since its eligibility was established, but outcome data were unavailable, it would be reclassified directly as *included without analysable outcome data*, regardless of its formal completion status.

The reclassification of the seven ongoing studies (Table 3) offers an equally clear illustration of the framework's advantage. Two were completed and being analysed at the time of the review (NCT03377504; NCT03887962). Under the current system, both were classified as ‘ongoing’, a status that is ambiguous given that data collection had concluded but results were not yet published. Under the revised framework, since their eligibility was established and analysable outcome data were not yet available, both would be reclassified as *included without analysable outcome data*. This reclassification is consistent with the empirical finding that the concept of ‘ongoing’ lacks a consistent definition across registries and guidelines [4], and that being ‘in analysis’ does not correspond clearly to a defined completion status. The remaining five ongoing studies (two actively in progress, one delayed and two of unknown status) had established eligibility and no available outcome data. All five would therefore also be classified as *included without analysable outcome data*, based on data availability rather than formal study status.

This example demonstrates that the proposed framework does not require additional primary data collection, as it operates on information already available to review authors. Its main effect is to impose a clearer decision logic (eligibility first, data availability second) that resolves the ambiguities currently absorbed into the ‘awaiting’ and ‘ongoing’ categories.

## Author Contributions

Conception and design: Rafael Leite Pacheco and Rachel Riera. Drafting the work: Rafael Leite Pacheco. Revising content: Rachel Riera. Final approval: all authors.

## Funding

The authors have nothing to report.

## Ethics Statement

The authors have nothing to report.

## Conflicts of Interest

The authors declare no conflicts of interest.

## Data Availability

Data sharing is not applicable to this article as no data sets were generated or analysed during the current study.
